# Hydration and symptoms in the last days of life

**DOI:** 10.1136/bmjspcare-2018-001729

**Published:** 2019-08-31

**Authors:** Martine E Lokker, Agnes van der Heide, Wendy H Oldenmenger, Carin C D van der Rijt, Lia van Zuylen

**Affiliations:** 1 Public Health, Erasmus MC, Rotterdam, The Netherlands; 2 Medical Oncology, Erasmus Medical Center, Rotterdam, The Netherlands

**Keywords:** end of life care, hydration, symptoms, death, dying

## Abstract

**Objectives:**

At the end of life oral fluid intake is often reduced. Consensus about the most appropriate management for terminally ill patients with limited oral fluid intake is lacking. The objective of this study is to investigate to what extent the amount of fluid intake, preceding and during the dying phase, is related to the occurrence of death rattle and terminal restlessness.

**Methods:**

A multicentre prospective observational study was performed. Data on the occurrence of death rattle and terminal restlessness, fluid intake and opioid use of patients expected to die within a few days or hours were collected.

**Results:**

371 patients were included. Death rattle was reported at least once in 40% (n=149) of patients during the dying phase. Death rattle occurrence was not associated with the amount of fluid intake during the days before dying. Terminal restlessness was reported in 26% of patients (n=96). Terminal restlessness was not associated with a lower amount of fluid intake during the days before dying. Terminal restlessness during the last 24 hours of life was associated with a higher amount of fluid (ie, >250 mL/day) during 48–25 hours before death.

**Conclusions:**

Caution with fluid intake to prevent development of death rattle does not seem to be necessary. Our study suggests that a higher amount of fluid intake during 48–25 hours before death may be associated with the occurrence of terminal restlessness during the last 24 hours of life. These results suggest that actively providing dying patients with artificial fluid may not be beneficial.

## Introduction

Most patients with a deteriorating chronic illness have a reduced oral intake at the end of life. This may be due to illness or treatment-related symptoms or complications, such as dysphagia, nausea or vomiting, generalised weakness and, in the last days of life, to a decreased level of consciousness or a loss of desire to drink.^1 2^ The evidence that artificial hydration (AH) may be beneficial when patients have a reduced oral intake in the last days of life is limited and inconclusive.^3–5^ Common arguments against AH are that it may increase the risk of complications such as oedema, ascites and death rattle.^1 6 7^ On the other side, the most commonly mentioned benefits of AH are that hydration may alleviate patients’ feelings of thirst and reduce the risk of delirium or terminal restlessness.^1 5 6 8–10^ Opinions vary on whether or not AH prolongs the dying process.^1 5 11 12^ As a result of these opposing arguments, attitudes whether or not AH should be used at the end of life vary among professional caregivers.^5–7 13^ Professional caregivers working in palliative care tend to be more reserved about the benefits of AH than other professionals: most of them do not believe that hydration contributes to the alleviation of symptoms or prolongs survival.^10 13^ Moreover, many of them are concerned about the additional burden of AH in the last week of life.^10 13^

The debate about possible benefits of AH has focused especially on two distinct symptoms in particular: death rattle and terminal restlessness. Death rattle due to respiratory tract secretion is a common symptom with a prevalence of 35% among dying patients and has been linked to overhydration at the end of life.^5 14 15^ Terminal restlessness, an agitated delirium at the end of life, is a common indication for palliative sedation and has been linked to underhydration at the end of life.^5 7 8 16–18^ The aim of this study is to investigate to what extent the amount of fluid intake, preceding and during the dying phase, is related to the occurrence of death rattle and terminal restlessness. The dying phase is defined as the phase when death is expected to occur within hours or days.^19 20^

## Methods

### Study design and population

We performed a multicentre prospective observational study in patients, 18 years or older, who were, according to the multidisciplinary care team, likely to die within a few days. Data were collected in eight hospitals (one to three wards per hospital) and five hospices, including three palliative care units in nursing homes, in the Netherlands. Data collection took place between November 2012 and November 2013.

### Data collection

Anonymous data were collected using a digital version of the Care Program for the Dying (CPD), a Dutch instrument for multidisciplinary care for patients in the dying phase that was originally based on the Liverpool Care Pathway for the dying patient.^21^ It was adapted to the Dutch language and healthcare system. The CPD is started when the multidisciplinary team agrees that the patient is likely to die within a few days and consists of three parts in which different data are recorded by nurses and physicians. For this study, the CPD was supplemented with questions about death rattle, terminal restlessness, patients’ fluid intake and use of opioids. We used the following data from the first part of the CPD, reflecting patient characteristics and the patient’s situation at the start of the CPD: diagnosis (cancer, non-cancer), gender, date of birth, date and time of the start of the CPD, level of consciousness (conscious, semiconscious, unconscious) and prevalence of restlessness, confusion and respiratory tract secretions. Further, we used data from questions that were added to part 1, about patients’ opioid use during the last day before the start of the CPD, and their total fluid intake (oral, intravenous and via feeding tube) during the last week and during the last day before the start of the CPD. Questions that were added to the second part of the CPD, reflecting the patient’s situation from the start of the CPD until death, concerned total fluid intake, opioid use, occurrence of death rattle and terminal restlessness, all per four hourly intervals. From the third part of the CPD, reflecting the situation after the patient’s death, we used data about the date and time of the patient’s death and the provision of sedation during the dying phase. See the online supplementary appendix for a detailed description of the variables that were used for this study.

10.1136/bmjspcare-2018-001729.supp1 Supplementary data

### Analysis and statistics

Patients were excluded from the study if data were missing on the date and time of the start of the CPD, the date and time of death or for more than six consecutive measurements (CPD, part 2).

In this study, the dying phase was defined as the phase when death is expected to occur within hours or days.^19 20^ *Duration of the dying phase* was calculated using the start date and time of the CPD and the date and time of the patient’s death. *Total fluid intake* was based on the intake of oral fluid, intravenous fluid as well as fluid intake via a feeding tube. Nurses estimated patient’s oral intake during the week before the start of the CPD, during the last day before the start of the CPD and during the dying phase. The fluid intake by different routes was added per period of time and scored into three categories: 0–499 mL/day, 500–999 mL/day and ≥1000 mL/day for the periods preceding the start of the CPD; and 0–249 mL/day, 250–499 mL/day and ≥500 mL/day during the dying phase. *Opioid doses* were recalculated to the morphine equivalent daily dose (mg/day) according to published equianalgesic dose tables;^22 23^ oral morphine 60 mg/day=parental morphine 20 mg/day=transdermal/parenteral fentanyl 25 µg/hour=oral oxycodone 40 mg/day=parental hydromorphone 4 mg/day=transdermal buprenorphine 26 µg/ hour.

**Figure 1:**
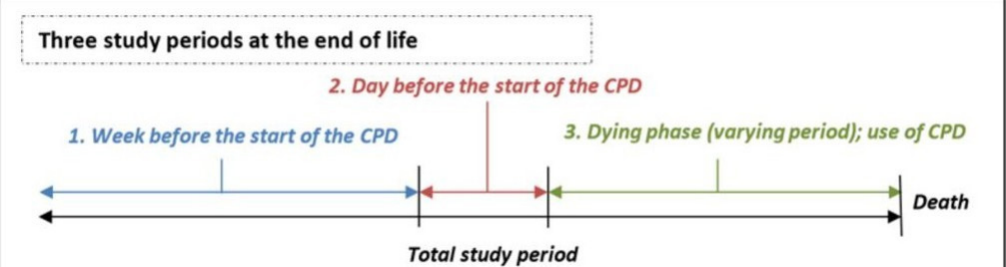
Data collection in relation to the moment of dying. CPD, Care Program for the Dying.

In case the dying phase had a duration longer than 48 hours, *fluid intake and opioid dose during 48–25 hours before death* (ie, the last day preceding the last 24 hours of life) were calculated using the four hourly consecutive measurements from part 2 of the CPD. In case the dying phase had a duration between 24 and 48 hours, fluid intake and opioid dose were calculated using data concerning the intake/dose during the last day before the start of the CPD and the four hourly information from the consecutive measurements from part 2 of the CPD. In case the dying phase had a duration shorter than 24 hours, fluid intake and opioid dose were based on the data concerning the intake/dose during the last day before the start of the CPD (figure 1). *Death rattle* occurrence was assessed by the attending nurse using the scoring scale as proposed by Back *et al*.^24^ This scoring scale records the experienced volume of death rattle: 0—inaudible; 1—audible only very close to the patient; 2—clearly audible at the end of the bed, in a quiet room; 3—clearly audible at the door of the room (about 20 feet/10 m), in a quiet room. In this study a patient was considered to have death rattle when there was a score of ≥2 on at least one four hourly measurements during the dying phase. *Terminal restlessness* occurrence was assessed by the attending nurse using the calmness scale of the Vancouver Interaction and Calmness Scale (VICS).^25^ The calmness scale consists of five items: (1) patient appears calm; (2) patient appears restless; (3) patient appears distressed; (4) patient is moving around uneasily in bed; and (5) patient is pulling at lines/tubes. Each item is scored on a 6-point Likert scale (strongly agree, agree, mildly agree, mildly disagree, disagree, strongly disagree). In this study, a patient was considered to experience terminal restless when, focusing on statements 2–5, two statements were scored with ‘strongly agree’ or ‘agree’ on at least one four hourly measurements, or when at least one statement was scored with ‘strongly agree’ or ‘agree’ on at least two consecutive measurements.

The primary outcome of this study was the association between death rattle or terminal restlessness and patients’ fluid intake during the week and day before the start of the CPD and during 48–25 hours before death. The statistical significance of this association was tested using the non-parametric Mann-Whitney tests. The secondary outcomes were associations between death rattle or terminal restlessness and other characteristics, including patients’ gender, age at death, diagnosis, place of death, duration of the dying phase, level of consciousness at the recognition of the dying phase and opioid use (ie, during the day before the start of the CPD and during 48–25 hours before death). These were analysed using Chi-Square test or Mann-Whitney test, where appropriate. Non-parametric tests were used when data were not normally distributed. All analyses were performed using SPSS for Windows V.22.0 (SPSS).

## Results

### Patient characteristics

**Figure 2:**
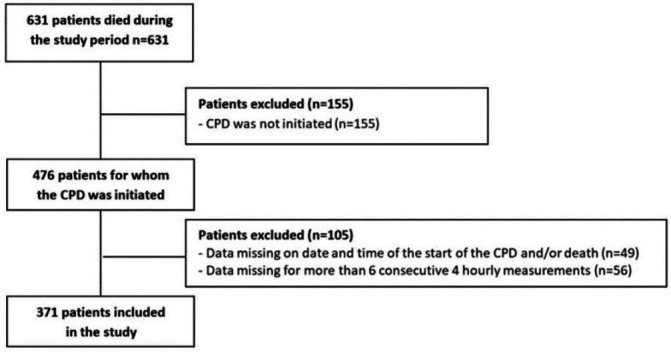
Flow chart. CPD, Care Program for the Dying.

During the study period 631 patients died in the participating care settings. The CPD was initiated for 476 patients (75% of all deaths), 371 of whom were included in this study (59% of all deaths). One hundred and five patients could not be included; 49 due to missing data about the date and time of the start of the CPD and/or death and 56 due to missing data for more than six consecutive four hourly measurements (part 2 of the CPD) (figure 2).

The included patients had a mean age of 72 years, almost half of them were male and 79% had been diagnosed with cancer (table 1). Forty-four percent of patients died in the hospital, 56% in the hospice. The median duration of the dying phase was 25 hours for all patients, 23 hours for patients dying in a hospital and 29 hours for patients dying in a hospice. Twenty-eight percent of patients had a duration of the dying phase longer than 48 hours, 28% had a duration between 24 and 48 hours and 44% had a duration shorter than 24 hours. At the start of the CPD, 22% of the patients were unconscious, 36% were restless, 24% were confused and 19% presented with respiratory tract secretions. Seventy-six percent of the patients used opioids during the last day before the start of the CDP and 93% during the dying phase.

**Table 1:** Patient characteristics (n=371)

	n (%)
Gender	
Male	181 (49%)
Female	190 (51%)
Age at death (years: mean, SD)	72 (14)
Diagnosis	
Cancer	289 (79%)
Non-cancer	76 (21%)
Place of death	
Hospital	164 (44%)
Hospice	207 (56%)
Duration of the dying phase (hours: median, range)	25, 0–279
*Symptoms at the start of the CPD*	
Level of consciousness	
Conscious	112 (30%)
Semiconscious	175 (47%)
Unconscious	80 (22%)
Restlessness	132 (36%)
Confusion	86 (24%)
Respiratory tract secretions	68 (19%)
*Treatment*	
Opioid use during the last day before the start of the CPD	282 (76%)
Morphine equivalent daily dose (mg/24 hours) (median, range)	75 (2–4200)
Opioid use during the dying phase	345 (93%)
Morphine equivalent daily dose (mg/24 hours) (median, range)	108 (0–10 790)
Use of palliative sedation during the dying phase	162 (44%)
*Fluid intake*	
Total daily fluid intake last week before start of the CPD (mL) (median, range)	625 (125–3375)
0–499 mL/day	74 (22%)
500–999 mL/day	117 (35%)
≥1000 mL/day	144 (43%)
Total daily fluid intake last day before start of the CPD (mL) (median, range)	625 (125–2875)
0–499 mL/day	187 (55%)
500–999 mL/day	70 (21%)
≥1000 mL/day	81 (24%)
Total daily fluid intake during the dying phase (mL) (median, range)	250 (6–2250)
0–249 mL/day	257 (69%)
250–499 mL/day	61 (16%)
≥500 mL/day	53 (15%)
Total daily fluid intake 48–25 hours before death (mL) (median, range)	334 (42–2500)
0–249 mL/day	190 (51%)
250–499 mL/day	44 (12%)
≥500 mL/day	121 (33%)

CPD, Care Program for the Dying.

### Fluid intake

Patients’ total fluid intake decreased during the last phase of life (table 1). During the week before the start of the CPD, 78% of patients had a total fluid intake of ≥500 mL/day, which decreased to 45% of patients during the last day before the start of the CPD and 15% during the dying phase. Fluid intake mainly involved oral intake, which decreased during the last days of life. During the week before the start of the CPD, 81% of patients had an oral intake of fluid, which decreased to 69% of patients during the last day before the start of the CPD and 41% during the dying phase. Twenty-four percent of patients had intravenous hydration during the week before the start of the CPD, 25% during the last day before the start and 34% during the dying phase. Intravenous hydration was predominantly prescribed in the hospital. Intake via a feeding tube involved 6% of patients during the week before the start of the CPD, 5% during the last day before the dying phase and 3% during the dying phase. Detailed information concerning fluid intake per care setting is described in table 2.

**Table 2:** Detailed information concerning fluid intake during the last period of life

	Total (n=371)	Hospital (n=164)	Hospice (n=207)
Week before the start of the CPD			
Orally			
n (%) Yes	302 (81%)	121 (74%)	181 (87%)
Median, range	625 mL (125–1000 mL)	625 mL (125–1000 mL)	625 mL (125–1000 mL)
Intravenous			
n (%) Yes	90 (24%)	85 (52%)	5 (2%)
Median, range	1250 mL (250–1500 mL)	1250 mL (250–1500 mL)	750 mL (750–750 mL)
Tube			
n (%) Yes	23 (6%)	18 (11%)	5 (2%)
Median, range	750 mL (250–1500 mL)	750 mL (250–1500 mL)	750 mL (250–1500 mL)
Day before the start of the CPD			
Orally			
n (%) Yes	255 (69%)	106 (65%)	149 (72%)
Median, range	125 mL (125–1000 mL)	625 mL (125–1000 mL)	125 mL (125–1000 mL)
Intravenous			
n (%) Yes	94 (25%)	90 (55%)	4 (2%)
Median, range	750 mL (250–1500 mL)	750 mL (250–1500 mL)	750 mL (250–750 mL)
Tube			
n (%) Yes	19 (5%)	17 (10%)	2 (1%)
Median, range	750 mL (250–1500 mL)	750 mL (250–1500 mL)	500 mL (250–750 mL)
During the dying phase			
Orally			
n (%) Yes	152 (41%)	66 (40%)	86 (42%)
Median, range	250 mL (25–1650 mL)	250 mL (25–1650 mL)	220 mL (27–1038 mL)
Intravenous			
n (%) Yes	125 (34%)	121 (74%)	4 (2%)
Median, range	179 mL (6–1500 mL)	179 mL (6–1500 mL)	297 mL (31–719 mL)
Tube			
n (%) Yes	12 (3%)	9 (6%)	3 (1%)
Median, range	21 mL (4–107 mL)	31 mL (5–107 mL)	9 mL (4–11 mL)

CPD, Care Program for the Dying.

### Prevalence of death rattle and terminal restlessness

**Figure 3:**
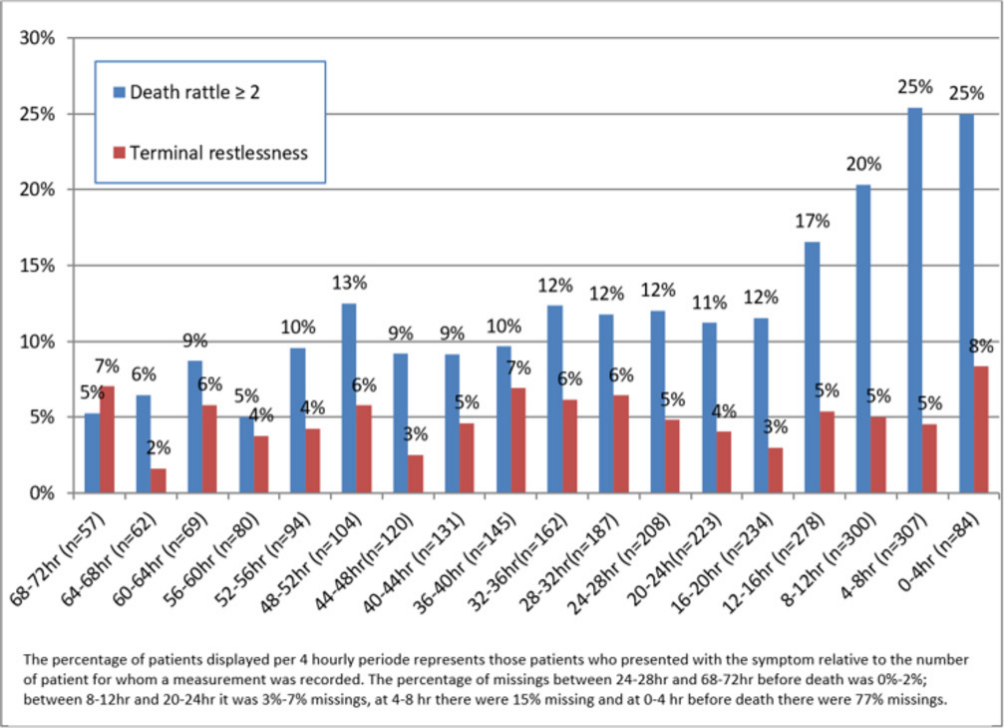
Percentage of patients with death rattle or terminal restlessness score per four hourly measurements during the last 72 hours of life.

Figure 3 shows the percentages of patients with death rattle or terminal restlessness per period of 4 hours before death. Overall, death rattle was reported at least once in 40% (n=149) of patients during the dying phase, and in 35% (n=130) of patients during the last 24 hours of life. Death rattle scores of ≥2 were often reported more than once; 62% of patients with death rattle had two or more death rattle scores of ≥2% and 35% of these patients had three or more of such episodes (not listed in figure 3). The prevalence of death rattle increased with death coming nearer. Terminal restlessness was reported at least once in 26% of patients (n=96) during the dying phase and in 13% (n=49) of patients during the last 24 hours of life. For most patients with terminal restlessness (61%), terminal restlessness was only reported once; 19% had terminal restlessness at ≥3 measurements (not listed in figure 3). Terminal restlessness occurred almost evenly throughout the dying phase.

### Associations between death rattle or terminal restlessness with fluid intake and other patient characteristics

No significant association between death rattle and the amount of fluid intake was found (table 3). A higher amount of fluid intake during the week and day before the start of the CPD tended to be associated with an increased occurrence of death rattle. Terminal restlessness was not associated with a lower amount of fluid intake during the days before dying. Terminal restlessness during the last 24 hours of life was statistically significantly associated with a higher amount of fluid during the time period of 48–25 hours before death (p=0.049). Patients’ level of consciousness at the start of the dying phase and the duration of the dying phase were also associated with terminal restlessness. Being conscious or semiconscious at the start of the dying phase was associated with a higher occurrence of terminal restlessness during the dying phase (p=0.004). A longer duration of the dying phase was also associated with a higher occurrence of terminal restlessness (p=0.003).

**Table 3:** Associations between death rattle or terminal restlessness with fluid intake and other patient characteristics (n=371)

	n	Symptom present somewhere during the dying phase				Symptom present during the last 24 hours of life			
		Death rattle score ≥2 (n=149)	P value	Terminal restlessness (n=96)	P value	Death rattle score ≥2 (n=130)	P value	Terminal restlessness (n=49)	P value
		n (%)		n (%)		n (%)		n (%)	
Gender			0.361		0.323		0.575		0.342
Male	181	77 (43)		51 (28)		66 (36)		27 (15)	
Female	190	72 (38)		45 (24)		64 (34)		22 (21)	
Age (years)			0.51		0.12		0.084		0.159
<65	102	44 (43)		33 (32)		40 (39)		19 (19)	
65–75	116	36 (31)		28 (24)		31 (27)		12 (10)	
>75	152	68 (45)		35 (23)		58 (38)		18 (12)	
Diagnosis			0.830		0.643		0.970		0.640
Cancer	289	118 (41)		76 (26)		102 (35)		36 (12)	
Non-cancer	76	30 (39)		18 (24)		27 (36)		11 (14)	
Place of death			0.977		0.117		0.748		0.679
Hospital	164	66 (40)		49 (30)		56 (34)		23 (14)	
Hospice	207	83 (40)		47 (23)		74 (36)		26 (13)	
Duration of the dying phase (hours)			0.458		**0.003**		0.416		0.138
<24	169	62 (37)		30 (18)		62 (37)		16 (9)	
24–48	104	45 (43)		31 (30)		39 (38)		18 (17)	
>48	98	42 (43)		35 (36)		29 (30)		15 (15)	
Level of consciousness at the start of the CPD			0.153		**0.001**		0.350		0.159
Conscious	112	39 (35)		34 (30)		34 (30)		19 (17)	
Semiconscious	175	78 (45)		53 (30)		67 (38)		23 (13)	
Unconscious	80	29 (36)		8 (10)		26 (32)		6 (8)	
Opioid use during the last day before the start of the CPD			0.820*		0.221*		0.378*		0.233*
First quartile (<29 mg/24 hours)	67	26 (39)		16 (24)		23 (34)		10 (15)	
Second quartile (29–74 mg/24 hours)	64	26 (41)		20 (31)		25 (39)		11 (17)	
Third quartile (75–179 mg/24 hours)	69	23 (33)		15 (22)		22 (32)		7 (10)	
Fourth quartile (≥180 mg/24 hours)	72	34 (47)		23 (32)		29 (40)		13 (18)	
Opioid use during the period of 48–25 hours before death			NA		NA		0.332*		0.132*
First quartile (<8 mg/24 hours)	91	NA		NA		25 (27)		7 (8)	
Second quartile (8–57 mg/24 hours)	94	NA		NA		37 (39)		14 (15)	
Third quartile (58–143 mg/24 hours)	91	NA		NA		29 (32)		11 (12)	
Fourth quartile (≥144 mg/24 hours)	95	NA		NA		39 (41)		17 (18)	
Total daily fluid intake during the week before the start of the CPD			0.186*		0.074*		0.324*		0.265*
0–499 mL/day	74	26 (35)		14 (19)		23 (31)		7 (9)	
500–999 mL/day	117	47 (40)		33 (28)		42 (36)		16 (14)	
≥1000 mL/day	144	64 (44)		45 (31)		55 (38)		22 (15)	
Total daily fluid intake during the last day before the start of the CPD			0.065*		0.384*		0.435*		0.398*
0–499 mL/day	187	70 (37)		46 (25)		65 (35)		22 (12)	
500–999 mL/day	70	28 (40)		24 (34)		24 (34)		11 (16)	
≥1000 mL/day	81	41 (51)		22 (27)		33 (41)		12 (15)	
Total daily fluid intake during the period of 48–25 hours before death			NA		NA		0.130*		0.049*
0–249 mL/day	190	NA		NA		74 (39)		19 (10)	
250–499 mL/day	44	NA		NA		15 (34)		8 (18)	
≥500 mL/day	121	NA		NA		37 (31)		21 (17)	

Statistics: Chi-Square test; Mann-Whitney test.

*Mann-Whitney test.

CPD, Care Program for the Dying; NA, not applicable.

## Discussion

We found no significant association between the amount of fluid intake and the occurrence of death rattle. We did not find an association between a lower amount of fluid intake and terminal restlessness either. Terminal restlessness during the last 24 hours of life was however associated with a higher amount of fluid intake during the time period of 48–25 hours before death.

Three previous studies have assessed death rattle occurrence and its association with AH.^7 9 14^ Morita *et al* 14 performed a multicentre, prospective, observational study of patients dying from cancer. Patients were divided into two groups: those who received 1 L or more of AH per day both 1 week and 3 weeks before death (hydration group, n=59) and those who did not (non-hydration group, n=167). During the last 3 weeks of life, 44% of patients in the hydration group and 46% of patients in the non-hydration group were recorded as presenting with death rattle (p=0.79). Yamaguchi *et al* 9 also performed a prospective observational study of patients dying from cancer. A comparison was made between patients who received more than 1 L of AH a day (large-volume hydration group, n=76) and patients who received less than 1 L/day (small-volume hydration group, n=75). However, this classification was made on the basis of their intake of AH at inclusion in the study, not taking into account any change in fluid intake closer to death. In total, 43% of patients were recorded as presenting with death rattle during 48 hours before death, 51% in the large-volume group and 35% in the small-volume group (p=0.07). Fritzson *et al* 7 performed a medical record review in which he studied patients who died in hospital and compared patients who had received parenteral fluid (PF group) with matched control patients who had not received parenteral fluid (non-PF group). During the last week of life 60% of all patients presented with death rattle, 63% in the PF group and 50% in the non-PF group (p=0.07). During the last 24 hours of life 46% of all patients presented with death rattle, 50% in the PF group and 33% in the non-PF group (p=0.02). Morita *et al* 14 and Fritzson *et al* 7 both reported on an AH intake of 1 L during the last 24 hours before death, not taking into account any oral intake. These intakes of AH are high compared with the median fluid intake in our study; 334 mL during the day preceding the last 24 hours.

We did not find an association between a lower amount of fluid intake during the days before dying and the occurrence of terminal restlessness. Terminal restlessness during the last 24 hours of life was however associated with a higher amount of fluid during the time period of 48–25 hours before death. Previous studies on the relation between fluid intake and occurrence of terminal restlessness or delirium showed diverse results. Morita *et al* 14 failed to show a difference in delirium occurrence between hydrated and non-hydrated patients with a prevalence of 12% in the hydration group and 13% in the non-hydration group (=0.80). Bruera *et al* 11 performed a randomised controlled trial in 129 patients with advanced cancer. Patients were divided into two groups: those who received 1 L of AH per day for the duration of a week (hydration group), and those who received 110 mL of AH per day (placebo group). No differences were found between the two groups in the occurrence of delirium. Yamaguchi *et al* 9 found a higher occurrence of delirium in patients who received less than 1 L of fluid a day compared with patients who received more than 1 L (17% vs 5%, p=0.01) and proposed hydration as an intervention-to-treat delirium. Our finding of an association between more fluid intake and more terminal restlessness is in line with the study by Fritzson *et al* 7 who also found a higher occurrence of terminal restlessness in patients receiving AH during the last 24 hours of life as compared with patients without AH.

Patients’ total fluid intake overall decreased during the last phase of life. However, in the hospital population the percentage of patients receiving parenteral fluids increased with death approaching. It is unclear why, but it is possible that healthcare professionals in hospital are more inclined or used to provide parenteral hydration at the end of life compared to professionals working in a hospice. It is also possible that both patient groups differed, eg in diagnosis. However, this did not result in a different occurrence of terminal restlessness and death rattle.

This study has some limitations. First, the optimal design to study the effects of fluid intake would be a randomised controlled trial, which would however pose ethical challenges. We conducted a prospective observational study to explore variations in fluid intake in daily practice. A recent study by Davies *et al* 26 indicates that a cluster randomised trial is feasible in the last days of life and therefore future studies should preferably use such a design. Second, the patient’s oral intake was based on nurses’ estimation. It would have been more reliable if we had used a fluid balance measure. However, a fluid balance measure could lead to medicalising the dying phase and is therefore not a common practice at the end of life. Third, at the start of this study no instrument to measure terminal restlessness was available. Because of the close connection between restlessness and calmness, we decided to use the calmness scale of the VICS. We did not use any other instrument to validate the calmness scale and it is possible that patients were misclassified. However, the prevalence reported in this study is in line with other studies focusing on terminal restlessness and delirium at the end of life. Fourth, we collected information on fluid intake at three moments in time: the week before the start of the CPD, the day before the start of the CPD and, four hourly, during the dying phase. Information on symptom occurrence was measured, four hourly, during the dying phase. Relating the total fluid intake during the dying phase to symptom occurrence during the dying phase could mean relating a symptom occurring at the start of the dying phase to an average level of fluid intake based on the entire dying phase. Therefore, we calculated a separate variable concerning the fluid intake during the time period of 48–25 hours before death. Whereas terminal restlessness occurred almost evenly throughout the dying phase and often only once, it is possible that patients in the non-symptom group during the last 24 hours of life actually presented with terminal restlessness before the last 24 hours of life but were successfully treated (ie, sedated). We found no evidence that patients with terminal restlessness were more often sedated compared with patients who were not terminal restless (p=0.23, not in table). Fifth, the high percentage of missings for the measurement period of 4–0 hours before death could mean that we overestimated or underestimated the occurrence of death rattle and terminal restlessness during that four hourly period. From daily practice we know that completing the measurement for this time period is often forgotten by nurses because completion is required after the patient has died. However, the terminal restlessness percentage is in line with what we would expect based on the other measurements and daily practice. Looking at the increase in death rattle occurrence during the four previous measurements, it is possible that the actual percentage for death rattle would have been higher. Consensus about the natural course of death rattle, whether it increases or decreases closer to death, is still lacking. Kåss and Ellershaw suggest that the prevalence of death rattle typically increases when death approaches.^27^ Yet, Heisler *et al* 28 performed a placebo-controlled trial and found a decrease of death rattle scores over time in the placebo group. Sixth, we did not make a distinction between types of opioids and added up opioids with different opioid metabolisms. It is possible that by combining opioids we lost the ability to show associations between specific opioid use and occurrence of symptoms on the one hand and/or specific opioid use and level of hydration on the other hand. Lastly, the observational design of the study precludes conclusions about causality.

In conclusion, we found that a higher amount of fluid intake (ie, possible overhydration), preceding and during the dying phase, was not associated with the occurrence of death rattle. Further, a lower amount of fluid intake (ie, possible underhydration), preceding and during the dying phase, was not associated with the occurrence of terminal restlessness. Caution with fluid intake to prevent development of death rattle does not seem to be necessary. Our study suggests that a higher amount of fluid intake during the period of 48–25 hours before death may be related to occurrence of terminal restlessness during the last 24 hours of life. These results suggest that actively providing dying patients with artificial fluid may not be beneficial, although intervention studies are needed to test this assumption.

## Data Availability

All data relevant to the study are included in the article or uploaded as supplementary information.
