# Supplementary material for: Hydration and symptoms in the last days of life
Source: BMJ Support Palliat Care. 2019 Aug 31;11(3):335–43. doi: 10.1136/bmjspcare-2018-001729 (PMC8380917; doi:10.1136/bmjspcare-2018-001729)
Supplement: Supplementary data [file bmjspcare-2018-001729supp001.pdf]

APPENDIX

Variables used in this study

Part 1 of the CPD, reflecting the patient's situation at the start of the dying phase
- Diagnosis (cancer, non-cancer)
- Gender (male, female)
- Date of birth
- Date and time of the start of the CPD
- Level of consciousness (conscious, semi-conscious, unconscious)
- Prevalence of restlessness (yes, no)
- Prevalence of confusion (yes, no)
- Prevalence of respiratory tract secretions (yes, no)

Questions added to part 1 of the CPD specifically for this study
- Has the patient used opioids in the last 24 hours? (yes/no)
    - Indicate route (transdermal, oral, rectal, oral, nasal), type (morphine,…) and the total dose during the past 24 hours
- Total fluid intake during the week preceding the recognition of the dying phase
    - Oral intake (1 cup is approximately 250 ml)
        - None
        - Sibs
        - Between 1-4 cups/ day
        - More than 4 cups/ day
    - Intravenous infusion
        - None
        - Less than 0,5 l/day
        - 0,5-1 l/day
        - 1-1,5 l/day
        - 1,5 l/day and more
    - Feeding tube
        - None
        - Less than 0,5 l/day
        - 0,5-1 l/day
        - 1-1,5 l/day
        - 1,5 l/day and more
- Total fluid intake during the last 24 hours preceding the recognition of the dying phase
    - Oral intake (1 cup is approximately 250 ml)
        - None
        - Sibs
        - Between 1-4 cups/ day
        - More than 4 cups/ day
    - Intravenous infusion
        - None
        - Less than 0,5 l/day
        - 0,5-1 l/day
        - 1-1,5 l/day
        - 1,5 l/day and more
    - Feeding tube
        - None
        - Less than 0,5 l/day
        - 0,5-1 l/day

- 1-1,5 l/day
- 1,5 l/day and more

Part 2 of the CPD, reflecting the patient's situation from the start of the dying phase until death

Questions added to part 2 of the CPD specifically for this study

- Total fluid intake, per four hourly intervals, until death
  - Oral intake (1 cup is approximately 250 ml)
    - None
    - Sibs
    - 1 cup
    - More than 1 cup
  - Intravenous infusion
    - None
    - Less than 0,5 l/day
    - 0,5-1 l/day
    - 1-1,5 l/day
    - 1,5 l/day and more
  - Feeding tube
    - None
    - Less than 0,5 l/day
    - 0,5-1 l/day
    - 1-1,5 l/day
    - 1,5 l/day and more
- Has the patient used opioids in the last 4 hours? (yes/no)
  - Indicate route (transdermal, oral, rectal, oral, nasal), type (morphine,…) and the total dose during the past 24 hours
- Has the patient had death rattle in the last 4 hours? (yes/no)
  - 0, inaudible
  - 1, audible only very close to the patient
  - 2, clearly audible at the end of the bed, in a quiet room
  - 3, clearly audible at the door of the room (about 20 feet / 10 meter), in a quiet room.
- Please indicate to what extend you agree (strongly agree, agree, mildly agree, mildly disagree, disagree, strongly disagree) with the following statements
  - 1. patient appears calm
  - 2. patient appears restless
  - 3. patient appears distressed
  - 4. patient is moving around uneasily in bed
  - 5. patient is pulling at lines/tubes.

Part 3 of the CPD, reflecting the situation after death

- Date and time of death

Questions added to part 3 of the CPD specifically for this study

- Did the patient receive palliative sedation (yes/no)
